# Comparative mitochondrial genome analysis reveals intron dynamics and gene rearrangements in two *Trametes* species

**DOI:** 10.1038/s41598-021-82040-7

**Published:** 2021-01-28

**Authors:** Cheng Chen, Qiang Li, Rongtao Fu, Jian Wang, Guangmin Deng, Xiaojuan Chen, Daihua Lu

**Affiliations:** 1 Institute of Plant Protection, Sichuan Academy of Agricultural Sciences, Key Laboratory of Integrated Pest Management On Crops in Southwest, Ministry of Agriculture, Chengdu, People’s Republic of China; 2 Key Laboratory of Coarse Cereal Processing, Ministry of Agriculture and Rural Affairs, School of Food and Biological Engineering, Chengdu University, Chengdu, People’s Republic of China; 3 Present Address: Sichuan Academy of Agricultural Sciences, 20 # Jingjusi Rd, Chengdu, 610066 Sichuan People’s Republic of China

**Keywords:** Fungi, Phylogenetics

## Abstract

*Trametes* species are efficient wood decomposers that are widespread throughout the world. Mitogenomes have been widely used to understand the phylogeny and evolution of fungi. Up to now, two mitogenomes from the *Trametes* genus have been revealed. In the present study, the complete mitogenomes of two novel *Trametes* species, *Trametes versicolor* and *T. coccinea*, were assembled and compared with other *Polyporales* mitogenomes. Both species contained circular DNA molecules, with sizes of 67,318 bp and 99,976 bp, respectively. Comparative mitogenomic analysis indicated that the gene number, length and base composition varied between the four *Trametes* mitogenomes we tested. In addition, all of the core protein coding genes in *Trametes* species were identified and subjected to purifying selection. The mitogenome of *T. coccinea* contained the largest number of introns among the four *Trametes* species tested, and introns were considered the main factors contributing to size variations of *Polyporales*. Several novel introns were detected in the *Trametes* species we assembled, and introns identified in *Polyporales* were found to undergo frequent loss/gain events. Large-scale gene rearrangements were detected between closely related *Trametes* species, including gene inversions, insertions, and migrations. A well-supported phylogenetic tree for 77 Basidiomycetes was obtained based on the combined mitochondrial gene set using 2 phylogenetic inference methods. The results showed that mitochondrial genes are effective molecular markers for understanding the phylogeny of Basidiomycetes. This study is the first to report the mitogenome rearrangement and intron dynamics of *Trametes* species, which shed light on the evolution of *Trametes* and other related species.

## Introduction

*Trametes* species, belonging to *Polyporales*, Basidiomycota, are widely distributed throughout the world^1^. *Trametes* species are often distributed on standing dead or fallen hardwood trees^2^. Like other species from the order *Polyporales*, *Trametes* species are efficient wood decomposers that cause white rot in colonized wood. Their efficient lignin decomposition ability makes *Trametes* important decomposers in ecosystems and plays an important role in the natural cycle of earth forest ecosystems. In addition, *Trametes* species have also been used in ex situ and in situ biosynthesis and bioremediation studies^3–6^. A series of lignin-degrading enzyme systems and genes have been found to be closely related to the excellent degradation ability of *Trametes*^7–9^, including lignin peroxidases, manganese peroxidases, and laccases. To date, only two mitochondrial genomes (mitogenomes) of *Trametes* species have been reported^10,11^, including *T. cingulata* and *T. hirsute*, and the characterizations and differentiation of mitogenomes in *Trametes* have not been fully analyzed.

Most eukaryotes contain mitogenomes, which are thought to be derived from Alphaproteobacteria through endosymbiosis^12,13^. The variation or mutation of mitogenomes can affect the growth, metabolism, and development of eukaryotes, leading to their aging and even death^14,15^. In addition, mitogenomes are also an effective tool to understand the evolution and phylogeny of eukaryotes^16,17^. Other characteristics of mitogenomes, such as genome size, intron type, tRNA genes, gene arrangement, and repeat sequences, have also become important references to reflect the evolutionary status of species^18–21^. The size, structure, and content of fungal mitogenomes vary greatly^22–24^, which has made it difficult to obtain complete fungal mitogenomes. When compared with the available fungal nuclear genome, the number of available fungal mitogenomes is very low (https://www.ncbi.nlm.nih.gov/genome/browse#!/overview/). The rapid development of high-throughput sequencing technologies, such as 454 pyrosequencing, Illumina (Solexa) sequencing, ABI SOLiD sequencing, Oxford Nanopore sequencing, and Pacbio SMRT sequencing, has promoted understanding of fungal mitogenomes.

In the present study, the mitogenomes of two *Trametes* species, *Trametes versicolor* and *T. coccinea*, were assembled and compared. The goals of this study were to: (1) characterize *Trametes* mitogenomes; (2) reveal the variations or similarities in genome size, structure, and gene content of the *Trametes* mitogenomes; (3) reveal the dynamic changes of introns in *Polyporales* mitogenomes; and (4) shed light on the phylogenetic relationships of *Trametes* in the phylum Basidiomycota based on the combined mitochondrial gene set. The results of this study will promote improved understanding of the origin, evolution, and taxonomy of *Trametes* species and other related fugal species.

## Results

### Features and PCGs of *Trametes* mitogenomes

**Figure 1:**
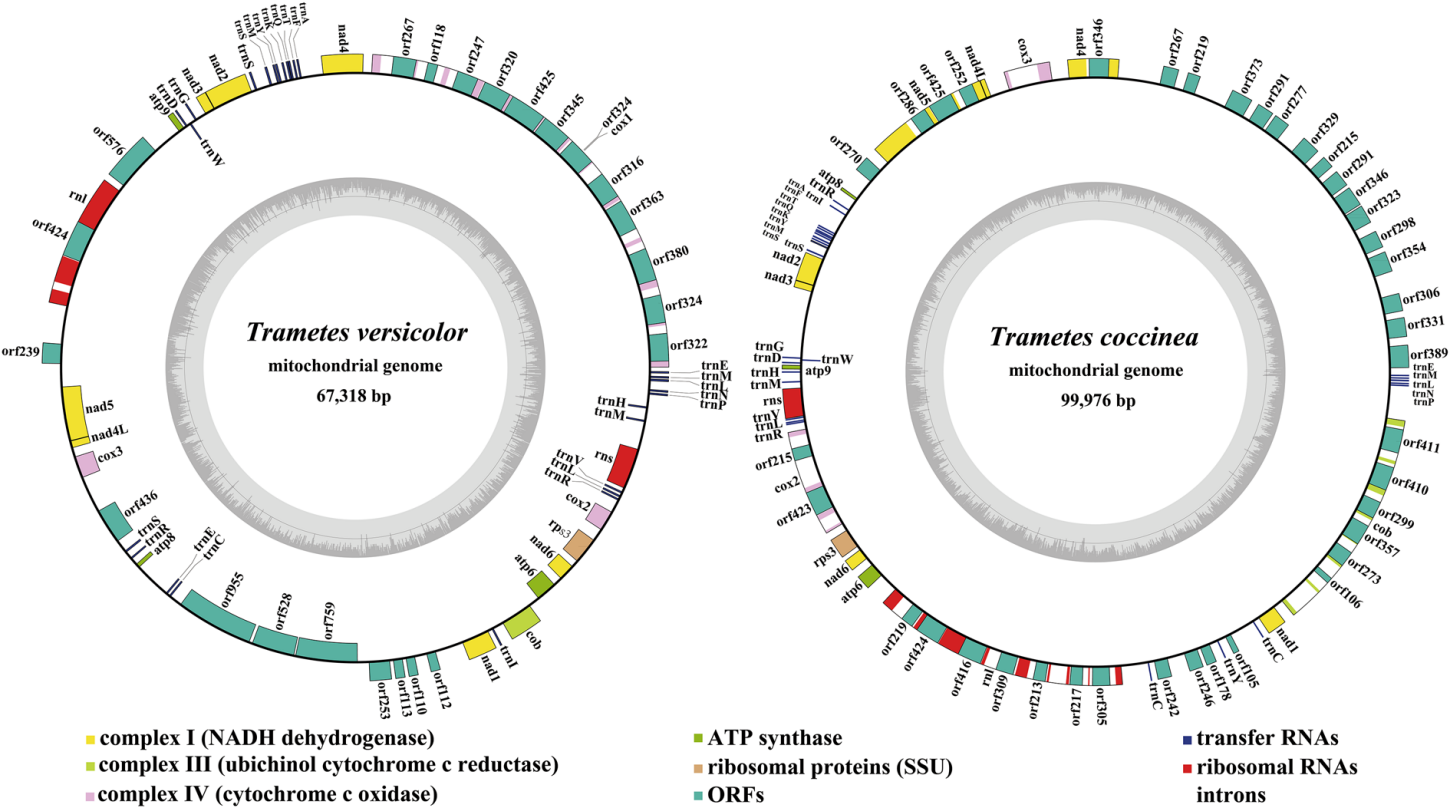
Circular maps of the two *Trametes* mitogenomes. Genes are represented by different colored blocks. Colored blocks outside each ring indicate that the genes are on the direct strand, while colored blocks within the ring indicates that the genes are located on the reverse strand.

The complete mitogenomes of *T. versicolor* and *T. coccinea* were circular with sizes of 67,318 bp and 99,976 bp, respectively (Fig. 1). The GC contents of the *T. versicolor* and *T. coccinea* mitogenomes were 25.44% and 24.68%, respectively (Table S1). Both the AT skew and GC skew in the *T. versicolor* mitogenome were positive. The mitogenome of *T. coccinea* contained negative AT skews and positive GC skews. We detected an entire set of core PCGs in the two *Trametes* mitogenomes we assembled, including *atp6*, *atp8*, *atp9*, *cob*, *cox1*, *cox2*, *cox3*, *nad1*, *nad2*, *nad3*, *nad4*, *nad4L*, *nad5*, *nad6*, and *rps3* (Table S2). In addition to these core PCGs, ten and five free-standing PCGs (non-intronic ORFs) were detected in the *T. versicolor* and *T. coccinea* mitogenomes, respectively. Several genes encoding DNA polymerase and RNA polymerase were detected in the two *Trametes* mitogenomes. In addition, the *T. versicolor* and *T. coccinea* mitogenomes contained seven and five PCGs with unknown functions, respectively. We also detected 14 and 38 introns in the *T. versicolor* and *T. coccinea* mitogenomes, respectively, which harbored 13 and 34 intronic ORFs. Intronic ORFs in the two *Trametes* mitogenomes mainly encoded LAGLIDADG endonucleases, GIY-YIG endonucleases, and intron-encoded RNA maturase bI4; however, we found two and three intronic ORFs with unknown functions in the *T. versicolor* and *T. coccinea* mitogenomes, respectively.

### rRNA genes and tRNA genes

Both the *T. versicolor* and *T. coccinea* mitogenomes contained two rRNA genes: the small subunit ribosomal RNA (*rns*) and large subunit ribosomal RNA gene (*rnl*) (Table S2). We detected two and eight introns in the *rnl* genes of *T. versicolor* and *T. coccinea*, respectively. The average size of the *rnl* gene was 3,013 bp, and the average length of *rns* genes in the two *Trametes* mitogenomes was 1,517 bp (excluding intron regions). The *T. versicolor* mitogenome contained longer *rnl* and shorter *rns* genes than the *T. coccinea* mitogenome.

The two *Trametes* mitogenomes we assembled each contained 27 tRNA genes (Table S2) that encoded 20 standard amino acids and ranged in length from 71 to 88 bp. The trnS gene contained the largest sizes among all tRNAs detected, mainly because of the large extra arm. Both mitogenomes of *T. versicolor* and *T. coccinea* contained two tRNAs that code for leucine and arginine with different anticodons, as well as three tRNAs encoding serine with different anticodons. The two mitogenomes also contained three tRNAs encoding methionine with the same anticodons. In addition, the *T. versicolor* mitogenome contained two *trnE* codons encoding glutamate, while the mitogenome of *T. coccinea* contained two *trnC* codons encoding cysteine.

### Repetitive elements in *Trametes* mitogenomes

We detected 38 and 45 intragenomic duplications in the *T. versicolor* and *T. coccinea* mitogenomes, respectively (Table S3), through BlastN searches of the two *Trametes* mitogenomes against themselves. The sizes of these intragenomic duplications ranged from 33 to 974 bp in the two *Trametes* mitogenomes and the pair-wise nucleotide similarities of these duplications were between 66.40 and 100%. The largest repeats were found in the coding regions of *orf425* and *orf424* in the mitogenome of *T. coccinea*, while the largest repeats in the *T. versicolor* mitogenome were observed in the intergenic regions between *nad5* and *orf270*, as well as between *atp8* and trnR*,* with each repeating sequence being 240 bp long. Repeat sequences identified by BlastN searches accounted for 4.45% and 8.29% of the *T. versicolor* and *T. coccinea* mitogenomes, respectively. We also identified 33 and 7 tandem repeats in the mitogenomes of *T. versicolor* and *T. coccinea*, respectively (Table S4), using Tandem Repeat Finder. The longest tandem repeat sequence, which had a size of 121 bp, was observed in the intergenic region between *orf110* and *orf112* in the mitogenome of *T. versicolor*. Tandem repeats accounted for 2.39% and 0.27% of the *T. versicolor* and *T. coccinea* mitogenomes, respectively.

We also conducted BlastN searches of the two *Trametes* mitogenomes against their published nuclear genomes to determine whether any gene fragments were naturally transferred between the mitochondrial and nuclear genomes (Table S5). We detected one and four aligned fragments between the mitochondrial and nuclear genomes of *T. versicolor* and *T. coccinea*, respectively. These aligned fragments ranged from 45 to 173 bp, with pair-wise nucleotide similarities ranging from 90.75% to 100%. The largest aligned fragment was observed in the intergenic region between *trnC* and *orf242* in the *T. coccinea* mitogenome. A total of 45 bp and 504 bp aligned fragments were detected in the *T. versicolor* and *T. coccinea* mitogenomes, respectively, indicating potential gene segment transfering events between mitochondrial and nuclear genomes may have occurred in the evolution of *Trametes*.

### Mitochondrial gene rearrangement in *Trametes* species

**Figure 2:**
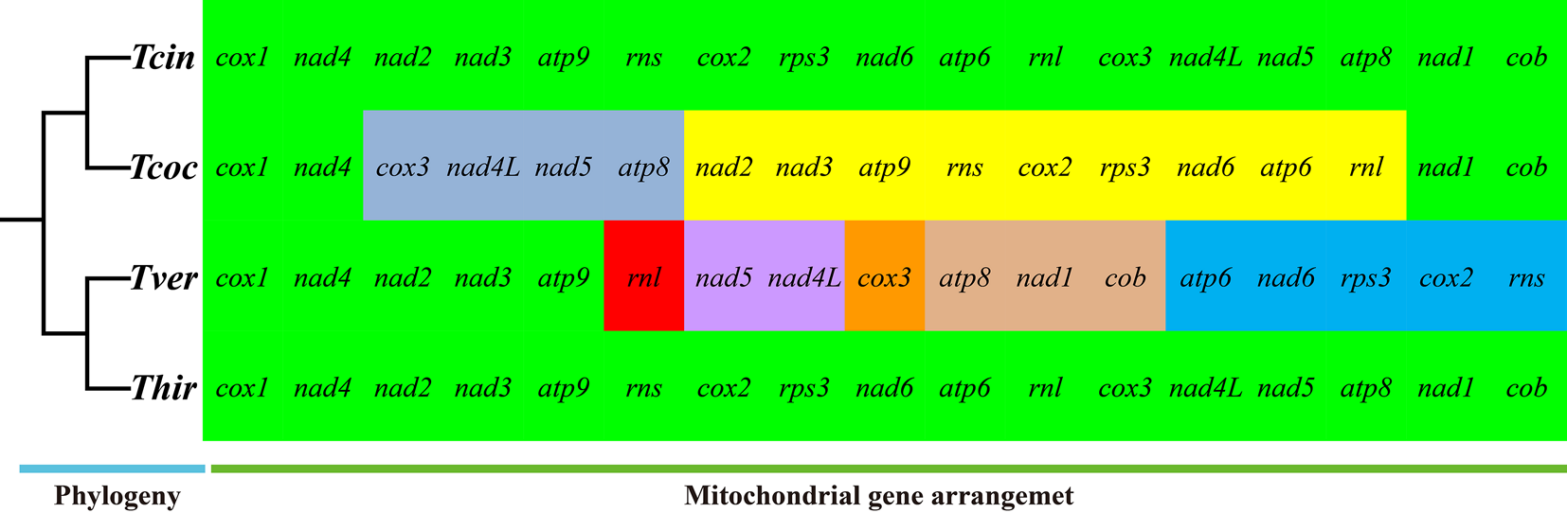
Gene order comparation between 4 *Trametes* species. All genes are shown in order of occurrence in the mitochondrial genome, starting from *cox1*. Fifteen core protein coding genes and two rRNA genes were included in the gene arrangement analysis. Genes with a green background indicate that they are conservative in Boletales species. Genes with other colored background indicate that they have gene rearrangements. The phylogenetic positions of 4 *Trametes* species were established using the Bayesian inference (BI) method and Maximum Likelihood (ML) method based on concatenated mitochondrial genes. Species and NCBI accession number used for gene arrangement analysis in the present study are listed in Supplementary Table S6.

We compared the arrangements of 15 core PCGs and 2 rRNA genes in the 4 *Trametes* mitogenomes reported in the present study (Fig. 2). *Trametes cingulata* and *T. hirsuta* were found to have an identical gene arrangement. We detected large-scale gene rearrangements in mitogenomes of *T. versicolor* and *T. coccinea* compared with the identical gene arrangement in *T. cingulata* and *T. hirsute*. These gene rearrangements included gene migrations, inversions, and insertions.

**Figure 3:**
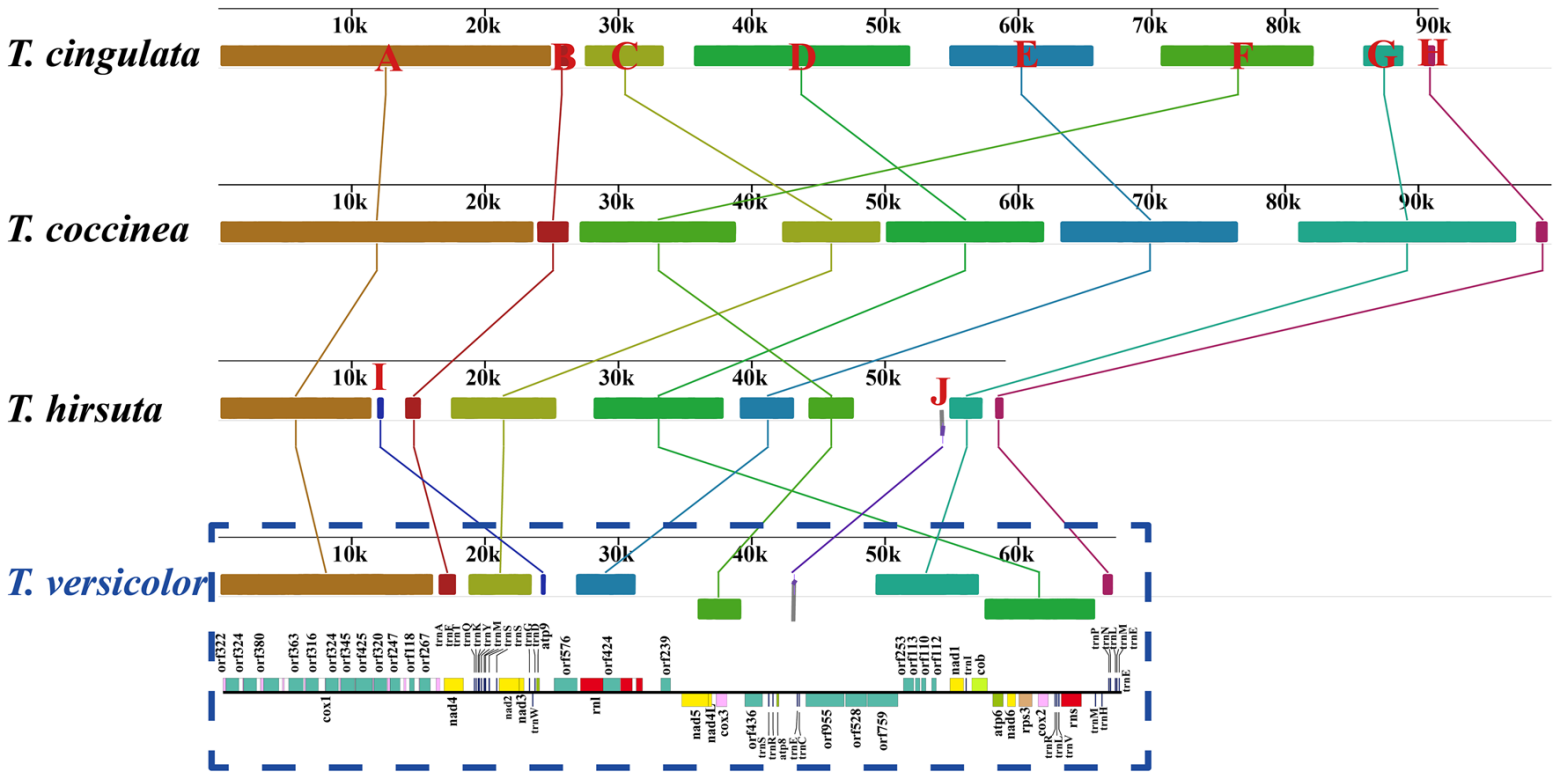
Mitochondrial gene collinearity analysis of 4 *Trametes* species using Mauve v2.4.0. Color blocks of the same color represent homologous regions between different mitogenomes. The schematic diagram of the mitogenome of *Trametes versicolor* is shown at the bottom of the picture.

Mauve^25^ revealed the presence of 10 homologous regions between the 4 *Trametes* species (Fig. 3), with different *Trametes* species found to contain various types and numbers of homologous regions. The *T. hirsuta* and *T. versicolor* mitogenomes contained additional homologous regions I and J, which involved the intergenic regions between neighboring genes *atp9* and orf576 and between *atp8* and trnE, respectively. Collinearity analysis indicated that the mitogenomes of *T. cingulata* and *T. hirsuta* were highly collinear, and that the *T. versicolor* and *T. coccinea* mitogenomes rearranged in homologous regions C, F, and D. The results indicated that the mitogenomes of *Trametes* underwent gene rearrangements in evolution.

### Variation, genetic distance, and evolutionary rates of core PCGs

**Figure 4:**
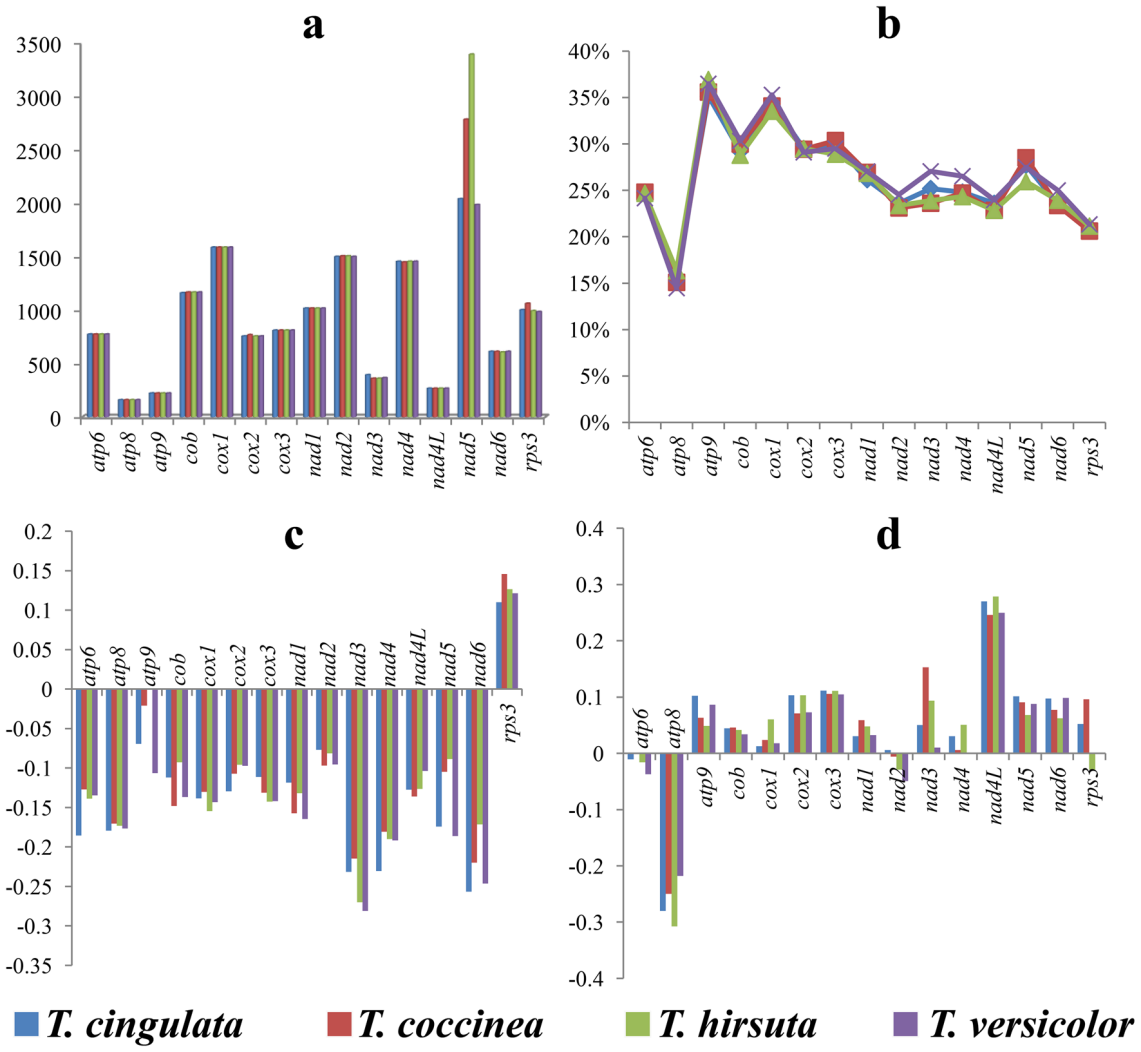
Variation in the length and base composition of each of 15 protein-coding genes (PCGs) between four *Trametes* mitogenomes. (**a**) PCG length variation; (**b**) GC content of the PCGs; (**c**) AT skew; (**d**) GC skew.

Seven of the 15 core PCGs detected (*cox2*, *nad2*, *nad3*, *nad4*, *nad5*, *nad6*, and *rps3*) varied in length between the 4 *Trametes* mitogenomes tested (Fig. 4). The *nad5* gene had the largest length variations between *Trametes* species, while the largest *nad5* gene was observed in *T. hirsute*. All of the 15 core PCGs except *atp6*, *cox2*, and *nad4L* had GC contents that varied between *Trametes* species. Among the 15 core PCGs detected, the *atp9* gene contained the highest GC content and the *atp8* gene contained the lowest. GC skews of the 15 core PCGs varied, with most showing positive GC skews.

**Figure 5:**
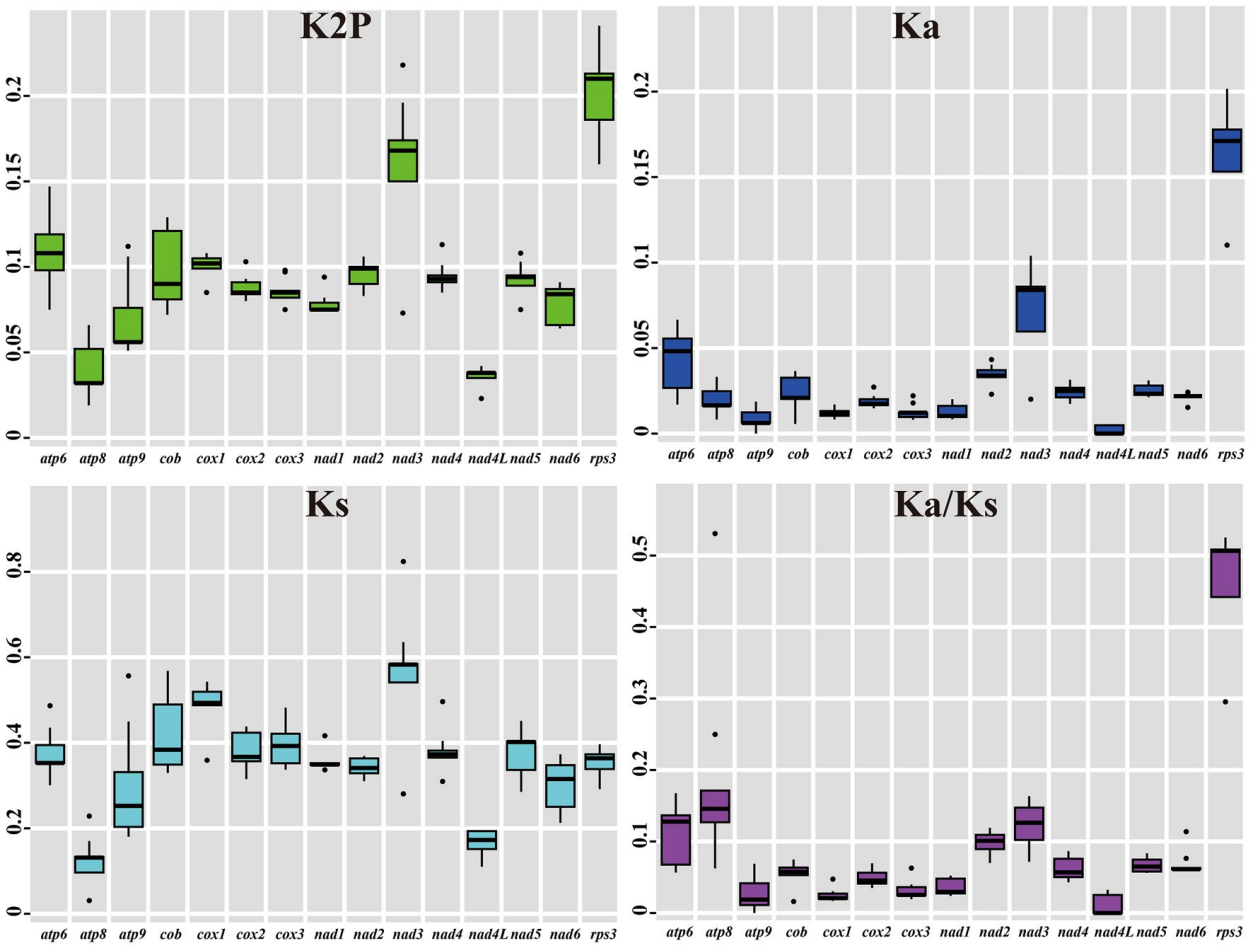
Genetic analysis of 15 protein coding genes conserved in the four *Trametes* mitogenomes. K2P, the Kimura-2-parameter distance; Ka, the mean number of nonsynonymous substitutions per nonsynonymous site; Ks, the mean number of synonymous substitutions per synonymous site.

The largest K2P genetic distance between *Trametes* species was observed in the *rps3* gene, followed by *nad3*, indicating the two genes differentiated greatly during evolution (Fig. 5). The *atp8* and *nad4L* genes exhibited the lowest mean K2P genetic distance between the four *Trametes* mitogenomes, indicating that they were highly conserved. The *rps3* gene exhibited the highest mean non-synonymous substitution (*Ka*) rate, whereas the *nad4L* gene had the lowest *Ka* mean value among the 15 core PCGs detected. We also found that the *nad3* gene had the highest mean synonymous substitution rate (*Ks*), whereas the *atp8* gene exhibited the lowest *Ks* mean value among the 15 core PCGs detected. The *Ka/Ks* values for all core PCGs were less than 1, indicating that the core genes in *Trametes* species were subjected to pressure of purifying selection.

### Intron dynamics of *cox1* gene in *Polyporales* species

**Figure 6:**
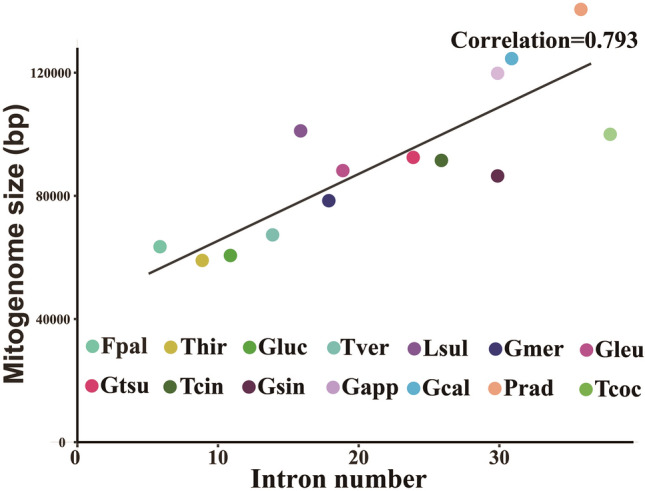
Pearson correlation analysis between the number of intron and mitogenome sizes of 14 Polyporales species. Species and NCBI accession numbers for genomes used in the phylogenetic analysis are provided in Supplementary Table S1.

Pearson correlation analysis showed that there was a high correlation coefficient between the number of introns and the size of the mitogenome in the order *Polyporales* (Fig. 6). The dynamics of introns in *Polyporales* had a significant effect on *Polyporales* mitogenome size. The *cox1* gene was found to be the largest host gene of introns in Basidiomycota^26,27^. In the present study, introns of *Polyporales cox1* genes were classified into different position classes (Pcls) using the *cox1* gene of *Ganoderma calidophilum* as a reference^22^, and introns belonging to the same Pcls were considered homologous introns. Overall, 153 introns were detected in the 14 *cox1* genes of the order *Polyporales*, and the number of introns in each *cox1* gene varied between 6 and 15, with an average of 11. Five of the 153 introns belonged to the group II, and the other introns belonged to the group I.

**Figure 7:**
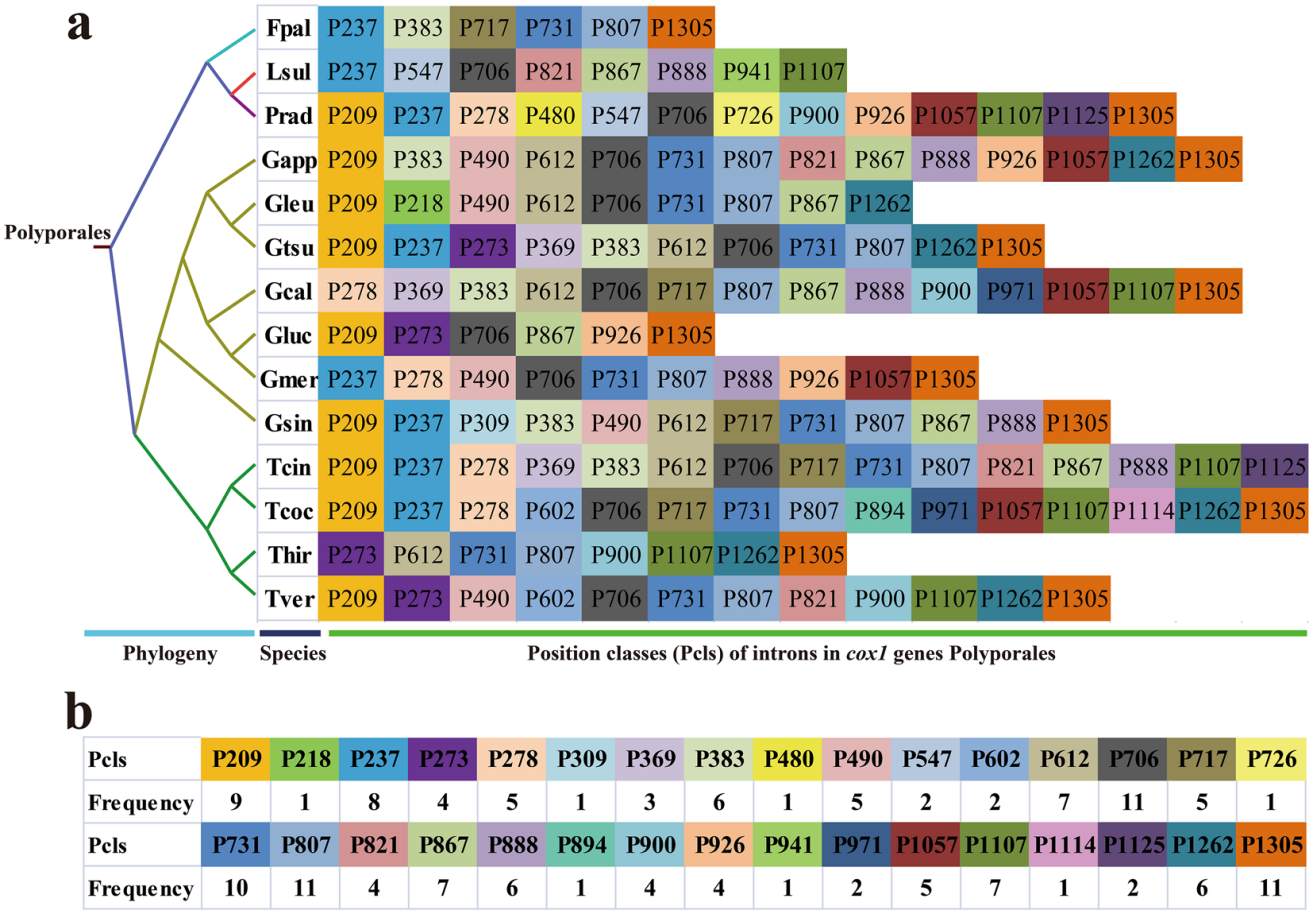
Position class (Pcl) information of *cox1* gene of the 14 Polyporales species. a, Pcls (orthologous introns) were named according to the insert sites (nt) in aligned reference *cox1* gene (MH252535). The phylogenetic positions of the 14 Polyporales species were established using the Bayesian inference (BI) method and Maximum Likelihood (ML) method based on combined mitochondrial gene set. b, the frequency of each Pcl in the 14 Polyporales species.

Overall, 32 Pcls were detected in the 14 *Polyporales* species (Fig. 7), with P706, P807, and P1305 being the most common intron types, occurring in 11 of the 14 *Polyporales* species. These commonly distributed introns may have been obtained from the common ancestor of *Polyporales* species. Seven of the 32 Pcls (P218, P309, P480, P726, P894, P941, and P1114) were only detected in one of the 14 *Polyporales* species. The rare introns in *Polyporales*, such as the P480 and P941, were also detected in distantly related species, such as *Armillaria sinapina*^28^ and *Agaricus bisporus*^29^ from the order *Agaricales*. These results indicated potential horizontal gene transfer events occurred during evolution. However, P218, P309, P276, P894, and P1114 were only distributed in *Polyporales*, and no homologous introns were found in other Basidiomycota species; accordingly, the origins of these rare introns need to be further investigated. P1107 was the most commonly distributed intron type in the four *Trametes* species we tested and was present in all four *Trametes* species. We detected two novel introns in our newly sequenced *T. coccinea* species, indicating diverse intron types in *Trametes* species. The varied classes and number of introns in *Trametes* species indicated that intron gain/loss occurred during evolution of the *Trametes* mitogenome.

### Comparative mitogenome analysis and phylogenetic analysis

The 99.976 kb mitogenome of *T. coccinea* was the largest mitogenome among the four *Trametes* species reported^10,11^ (Table S1). When compared with other *Polyporales* mitogenomes reported, the *Trametes* mitogenomes had medium sizes. The GC contents of the *Polyporales* species varied between 24.0 and 36.3%, with an average of 26.8%. Most *Polyporales* we detected contained positive AT and GC skews, indicating preferences for A and G bases in the leading strand of *Polyporales* mitogenomes. All *Polyporales* mitogenomes tested contained an entire set of core PCGs and a variety of free-standing ORFs. The mitogenome of *T. coccinea* contained the most introns among the 14 *Polyporales* species tested. Additionally, the highest number of intronic ORFs was detected in the mitogenome of *Phlebia radiata*^30^, which contributed to the *P. radiata* mitogenome becoming the largest mitogenome among the 14 *Polyporales* species we tested. All 14 *Polyporales* mitogenomes contained two rRNA genes, and the number of tRNA genes in the 14 *Polyporales* species ranged from 25 to 29.

**Figure 8:**
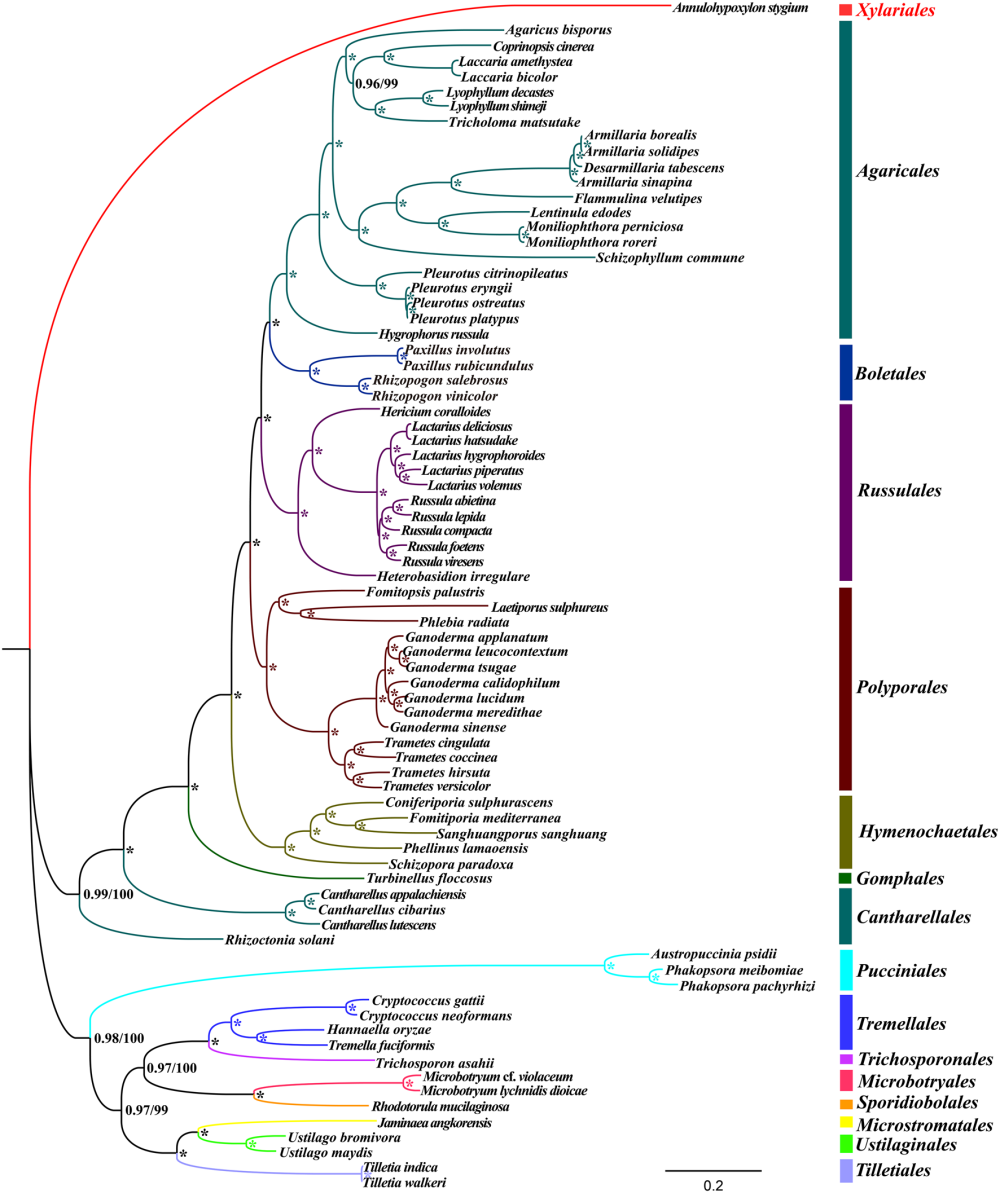
Phylogeny of 77 Basidiomycota species based on 15 protein coding genes and two rRNA genes using Bayesian inference (BI) and Maximum likelihood (ML) analysis. Support values are Bayesian posterior probabilities (before slash) and bootstrap (BS) values (after slash). The asterisk indicates that the BPP value is 1 and the BS value is 100 of the branch. Species and NCBI accession numbers for mitogenomes used in the phylogenetic analysis are provided in Supplementary Table S6.

An identical and well-supported phylogenetic tree was obtained using the maximum likelihood (ML) and Bayesian inference (BI) methods based on the combined mitochondrial gene set (15 core PCGs) (Fig. 8). All major clades within the phylogenetic tree were found to have good support values (BS ≥ 99; BPP ≥ 0.96). Overall, 77 Basidiomycota species were included in the phylogenetic analysis, and these were divided into 13 major clades corresponding to the orders *Agaricales*, *Boletales*, *Russulales*, *Polyporales*, *Hymenochaetales*, *Gomphales*, *Cantharellales*, *Pucciniales*, *Tremellales*, *Trichosporonales*, *Microbotryales*, *Sporidiobolales*, *Microstromatales*, *Ustilaginales*, and *Tilletiales* (Table S6). *T. coccinea* was a sister species to *T. cingulata* and *T. versicolor* was closely related to *T. hirsute*.

## Discussion

### Size and content variations between *Trametes* mitogenomes

In this study, two mitogenomes of the genus *Trametes*, *T. coccinea* and *T. versicolor*, were newly assembled. Comparative mitogenomic analysis showed that the novel mitogenome of *T. coccinea* was largest among the four *Trametes* mitogenomes reported^10,11^. Introns were considered the main factors contributing to the size expansion of the *T. coccinea* mitogenome, and the *T. coccinea* mitogenome was found to have the most introns among *Trametes* species. We also found a high correlation coefficient between the mitogenome size and the number of introns in *Polyporales*^22,30–35^. These findings further suggested that the dynamic change in introns is one of the main factors leading to the size variation of mitogenomes from the order *Polyporales* and other fungal species, which is consistent with the results of previous studies^29,36,37^. We also found that the mitogenome content of *Trametes* species varied. The four *Trametes* mitogenomes varied in AT and GC skews. According to the second parity rule, as long as there is no mutation or selection bias, each base in the complementary DNA strand exists at approximately equal frequencies^38^. The presence of AT or GC skews on the same DNA strand from different species indicated that mitogenomes of different *Trametes* species underwent different mutations or environmental selection^39^. In the present study, the core PCGs of *Trametes* species were found to vary frequently in length and base composition. Different core PCGs showed varied evolution rates, and some core PCGs were relatively conserved between *Trametes* species, whereas others showed large differentiation. All core PCGs were found to have been subjected to the pressure of purifing selection. In addition to these core PCGs, some non-conserved PCGs were also detected in *Trametes* species, including intronic ORFs, DNA polymerases, and RNA polymerases. Introns encoding endonucleases mainly mediate intron homing in *Trametes* mitogenomes^40^, while DNA and RNA polymerases are thought to be obtained from plasmids^41–43^. Several PCGs with unknown functions were also detected in *Trametes* species, indicating that there are still genes with unknown functions in *Trametes* mitogenomes that need to be revealed.

### Mitochondrial gene rearrangement in *Trametes* species

Mitochondrial gene rearrangement was frequently detected in mitogenomes of animals, plants, and fungi^18,44,45^. Variations in the mitochondrial gene order can be used to infer the phylogenetic status and phylogenetic relationship of eukaryotic species^46,47^. The rearrangement of the animal mitogenome has been widely studied, and several models have been proposed to reveal the rearrangement of animal mitogenomes^48,49^. When compared with the mitogenomes of animals, the mitogenomes of fungi show greater variation in gene order^50^. In the present study, we detected large-scale gene rearrangements between *Trametes* species, including gene inversions, insertions, and migrations. Gene rearrangement events have even been observed between closely related *Trametes* species within the same clades, indicating that the mitochondrial gene arrangement of *Trametes* species is highly variable. However, the mechanism of mitochondrial gene rearrangement in fungi has not been elucidated to date. Previous studies showed that the accumulation of repetitive sequences was the main factor contributing to mitochondrial gene rearrangement in fungi^50^. We found a large number of repeat sequences in *Trametes* species, which may result in gene recombination and rearrangement in these species.

### Intron dynamics of *Polyporales* species

Introns are considered mobile genetic elements in the eukaryote mitogenome, and their dynamic changes have a significant effect on the size and organization of mitogenomes^51–53^. Variations in mitochondrial introns among different eukaryote lineages vary greatly. Generally, animal mitogenomes do not contain any introns, whereas plant mitogenomes mainly contain group II introns and fungal mitogenomes mainly contain group I introns^51,54^. The *cox1* gene was found to be the main host gene of introns in mitogenomes of Basidiomycetes^55,56^. We divided the introns of *cox1* genes of 14 *Polyporales* species into different position classes (Pcls) according to their insertion sites in the protein coding region. The introns belonging to the same Pcl were considered homologous^29^. We found that the number and class of introns varied greatly between different *Polyporales* species, indicating that the loss/gain of introns occurred in the evolution of *Polyporales*. Several Pcls from common ancestors of *Polyporales*, such as P706, P807, and P1305, were found to be widely distributed in *Polyporales* species. However, several introns were found only in one of the 14 *Polyporales* species, such as P480 and P941, whereas homologous introns were detected in distant species^28,29^, indicating potential intron transfer events. We also found some novel introns in *Polyporales* that have never been detected in other Basidiomycetes, including P218, P309, P726, P894, and P1114. The origin, evolution, and function of these novel introns need to be further analyzed.

### Phylogeny of Basidiomycota species based on mitochondrial genes

Owing to the limited morphological characteristics, it is difficult to classify and identify Basidiomycetes accurately only by morphology. To date, only rRNA internal transcribed spacers (*ITS*)^57^, RNA polymerase II subunits (*RPB*), and elongation factor 1-ɑ (*EF1ɑ*) genes^58^ have been widely used to evaluate the phylogeny and reconstruct the early evolution of fungi. Mitochondrial genes have been widely used as molecular markers to analyze phylogenetic relationships of animals because of their unique advantages^16,44^. When compared with the mitogenomes of animals, the mitogenomes of fungi have been less studied, especially those of Basidiomycetes^59^. Currently, less than 130 complete mitogenomes of Basidiomycetes are available in the NCBI database (https://www.ncbi.nlm.nih.gov/genome/browse#!/overview/), which has limited comprehensive analysis of the origin and evolution of fungi. In this study, 77 Basidiomycete species were included in the phylogenic study. We have also obtained a phylogenetic tree with high support values based on the combined mitochondrial gene set, indicating that mitochondrial genes are a powerful tool for analyzing phylogenetic relationships of Basidiomycota species. With the rapid development of second and third generation sequencing technology, additional mitogenomes of fungi are needed to understand the origin and evolution of Basidiomycetes and other fungal lineages.

## Materials and methods

### Assembly and annotation of *Trametes* mitogenomes

The raw sequencing data used for *T. versicolor* and *T. coccinea* mitogenomes assembly were downloaded from the Sequence Read Archive (SRA) database under the accession numbers SRR3927404 and SRR1588030, respectively. We conducted a series of quality control steps to generate clean reads from the raw sequencing reads, including removing adapter reads using AdapterRemoval v2^60^ and filtering low-quality sequences using ngsShoRT 2.2^61^. We used the clean reads to assemble mitogenomes of *T. versicolor* and *T. coccinea* using SPAdes 3.9.0^62^. Several contigs were obtained using SPAdes 3.9.0^62^, and gaps between the contigs were filled using MITObim V1.9^63^. NOVOPlasty^64^ was also used to assemble or test mitogenomes of the two *Trametes* species. After these steps, we obtained the complete mitogenomes of *T. versicolor* and *T. coccinea*, and then annotated them according to previously described methods^33^. We initially annotated the protein-coding genes (PCGs), open reading frames (ORFs), introns, rRNA genes, and tRNA genes in the two *Trametes* mitogenomes using MFannot^65^ and MITOS^66^. PCGs or ORFs were also predicted based on the NCBI Open Reading Frame (ORF) Finder^67^ and annotated by BLASTP searches against the NCBI non-redundant protein sequence database^68^. Intron–exon borders of PCGs or ORFs were verified using Exonerate v2.2^69^. We also used tRNAscan-SE v1.3.1^70^ to predict or identify tRNA genes in the two *Trametes* mitogenomes. Finally, graphical maps of the two *Trametes* mitogenomes were drawn with OGDRAW v1.2^71^.

### Mitogenome sequence analysis

Base compositions of the two *Trametes* mitogenomes and other *Polyporales* mitogenomes were analyzed using DNASTAR Lasergene v7.1 (http://www.dnastar.com/). We calculated strand asymmetries of *Polyporales* mitogenomes using the following formulas: AT skew = [A – T]/[A + T] and GC skew = [G – C]/[G + C]^72^. We analyzed codon usages within the two *Trametes* mitogenomes using the Sequence Manipulation Suite^73^. We calculated the nonsynonymous (*Ka*) and synonymous (*Ks*) substitution rates for core PCGs in the four *Trametes* mitogenomes using DnaSP v6.10.01^74^. The genetic distances between each pair of the 15 core PCGs (*atp6, atp8, atp9, cob*, *cox1, cox2, cox3, nad1, nad2, nad3, nad4, nad4L, nad5, nad6,* and *rps3*) were detected using MEGA v6.06^75^ based on the Kimura 2-parameter (K2P) substitution model.

### Repetitive element analysis

We conducted BlastN searches of the two *Trametes* mitogenomes against themselves^76^ to determine whether there were intragenomic duplications of large fragments or interspersed repeats in the two *Trametes* mitogenomes. We also detected tandem repeats (> 10 bp in length) in the two *Trametes* mitogenomes using a Tandem Repeats Finder^77^ with the default parameters. In addition, BlastN searches of the two *Trametes* mitogenomes were conducted against their published nuclear genomes to determine whether there were gene segments that naturally transferred between mitochondrial and nuclear genomes^78,79^.

### Comparative mitogenomic analysis and intron analysis

The genome sizes, GC contents, base compositions, start and stop codons, gene numbers, and intron numbers of *Polyporales* mitogenomes were compared to identify variations and similarities between them. Introns of *cox1* genes in the 15 *Polyporales* mitogenomes that have been published to date were classified into different position classes (Pcls) according to the method described by Ferandon et al.^80^. The *cox1* genes of 15 *Polyporales* mitogenomes were first aligned with the *cox1* gene of the medical fungus *Ganoderma calidophilum*^22^ using Clustal W^81^. Each Pcl was composed of introns inserted at the same position of the *cox1* reference gene based on the insert sites (nt) in the corresponding reference gene. Identical Pcls were considered orthologous introns with high sequence similarity.

### Phylogenetic analysis

A phylogenetic tree of 77 Basidiomycota species was constructed to investigate the status of *Trametes* species in the phylum Basidiomycota using previously described methods^33,82^ based on the combined mitochondrial gene set (15 core PCGs). We used *Annulohypoxylon stygium* from the phylum Ascomycota as the outgroup^83^. Individual mitochondrial genes were first aligned using the MAFFT v7.037 software^84^, after which we concatenated the aligned mitochondrial genes into a combined mitochondrial gene set using the SequenceMatrix v1.7.8 software^85^. A preliminary partition homogeneity test was then conducted to detect potential phylogenetic conflicts between different genes. Best-fit models of partitioning schemes and evolution for the combined mitochondrial gene set were determined based on the PartitionFinder 2.1.1 software^86^. We constructed phylogenetic trees using both Bayesian inference (BI) and maximum likelihood (ML) methods, with MrBayes v3.2.6^87^ used for the BI analysis and RAxML v 8.0.0^88^ for the ML analysis.

### Data availability

The complete mitogenomes of *T. versicolor* and *T. coccinea* were deposited in the GenBank database under accession numbers MT479165 and MT479166, respectively.

## Supplementary Information

Supplementary Information.
